# Quality of life in patients with chordomas/chondrosarcomas during treatment with proton beam therapy

**DOI:** 10.1093/jrr/rrt057

**Published:** 2013-07

**Authors:** A. Srivastava, B. Vischioni, M.R. Fiore, V. Vitolo, P. Fossati, A. Iannalfi, J.K.L. Tuan, R. Orecchia

**Affiliations:** 1 Department of Radiation Oncology, Medanta the Medicity, Sector 38, Gurgaon 122001, Haryana, India; 2 Fondazione CNAO, Strada privata campeggi, Pavia, 27100, Italy; 3 National Cancer Centre Singapore, 11 Hospital Drive, Singapore 169610; 4 European Institute of Oncology, Via Ripamonti, 435, 20141 Milan, Italy

**Keywords:** quality of life, proton beam therapy, chordoma, chondrosarcoma

## Abstract

Introduction: Health-related quality of life (HQL) parameters have never been tested in patients having chondromas/chondrosarcomas who are being treated with protons. The aim of this study was to document changes in HQL of chordoma/chondrosarcoma patients treated with proton beam radiotherapy. Treatments commenced in September 2011 at CNAO, and HQL studies were initiated in January 2012 for all patients undergoing treatment. Methods: The validated Italian translation of the EORTC QLQ-C30 version 3.0 was used for HQL evaluation. The HQL assessments were made prior to starting radiation and at completion of treatment. Scoring was as per the EORTC manual. As per standard norms, a difference of >10 points in the mean scores was taken to be clinically meaningful. Results: Between January and September 2012, 17 patients diagnosed with chordoma or chondrosarcoma, with a mean ± SD age of 49.5 ± 16.4 years, had completed treatment. The involved sites were skull base (*n* = 12) and sacral/paraspinal (*n* = 5). The prescribed dose was 70–74 GyE at 2 GyE per fraction, 5 days/week. When comparing pre- and post-treatment scores, neither a clinically meaningful nor a statistically significant change was documented. Conclusions: During treatment, HQL is not adversely affected by protons, allowing normal life despite the long course of treatment. This is an ongoing study and more long-term assessment will help evaluate the actual impact of proton therapy on HQL for these slow-responding tumours.

## INTRODUCTION

In recent years, success or failure of a therapy for a specific disease was assessed in terms of overall survival, local control or complication rate. These objective parameters, assessed by the physician, formed the gold standard for evaluating and comparing outcomes. Though these did give an indication regarding the success or failure of the treatment methodology *per se*, they did not provide any information regarding the patient's mental and emotional wellbeing or the impact of the disease on the patient [1]. The treatment would be considered to be successful if the disease was controlled and survival prolonged, without considering the detrimental effects of the disease and the treatment on the daily life of the patient. Health-related quality of life (HQL) refers to the patient's perception of the effects of the disease and the impact on the patient's daily functioning. The survey (EORTC-QLQ C30ver 3.0) used in this study is multidimensional, incorporating all aspects of daily life, and it is subjective, i.e. self-reported by the patient [2, 3]. Recently, there has been a surge of interest in these aspects and more emphasis is being placed upon preserving a good quality of life (QOL) while improving/maintaining the objective measures of outcome, like disease control and survival.

Chordomas and chondrosarcomas are rare tumours that are usually slow growing with a tendency to be diagnosed at a relatively advanced stage, which usually present for radiotherapy after several resections have been attempted [4, 5]. They have a predilection to involve the appendicular skeleton and develop in close proximity to important anatomical structures like the optic apparatus, cranial nerves, brainstem, spinal cord and cauda equina, making surgical resection difficult. Complete surgical resection of these tumours offers the best control rates but is rarely achievable, and even after complete resections, there is a risk of microscopic residual disease that is at a high risk for recurrence [6]. Maximal surgical resection followed by radiotherapy has been found to have the best control rates, even though radiation alone itself has not been found to be very effective [7]. These are designated as relatively radioresistant tumours, and optimal doses of radiation are needed to effect local control [8, 9]. However, delivery of optimal doses with conventional techniques is difficult, and dose to the tumour needs to be scaled in keeping with the lower tolerance doses of the adjacent normal tissues.

Proton beam therapy is an emerging option that is expected to be superior to conventional photon therapy, primarily because of its ability to deposit optimal doses at the tumour site while limiting exposure of the adjacent normal tissues. This provides an opportunity to deliver higher doses to the tumour while respecting the tolerance of the normal tissues. Thus proton beam therapy can be expected to have the greatest possible survival with the fewest and least severe side effects [10, 11]. If this can be accomplished, then it is assumed that the QOL of the patients would be improved. However, it has to be emphasized that there also exists a possibility, though rare, that some types of side effects could be made worse due to incorrect relative biological effectiveness (RBE) allocation, Bragg peak positioning errors, or excess skin (entry) dose compared to photons, adversely affecting HQL. There is no published data on the quality-of-life changes in patients with chordomas or chondrosarcomas treated with proton beam therapy. This study aims to evaluate whether the HQL of these patients is affected during treatment with proton beam therapy. A prospective longitudinal evaluation of QOL of patients histologically diagnosed as having chordoma/chondrosarcoma of skull base or spine, who were treated at the Italian National Hadrontherapy Center for Cancer (CNAO) was performed, and the results of the same are discussed here.

## MATERIALS AND METHODS

### Study population

Proton therapy treatments at the Italian National Hadrontherapy Center (CNAO) at Pavia, Italy started in September 2011. A quality-of-life study of chordoma and chondrosarcoma patients was initiated in January 2012. Between January 2012 and July 2012, 17 patients had been treated within the approved protocols for chordoma or chondrosarcoma.

All patients were treated with proton beam therapy at the conventional fractionation of 2 GyE per fraction for 5 days a week to a total dose of 70 GyE for chondrosarcoma and 74 GyE for chordoma. The average treatment duration was 52.5 ± 4 SD days with a range of 45–59 days. There were no toxicity-related treatment breaks.

Additionally the attending physician scored the patient on four parameters *viz*. mobility, self-care, pain/discomfort and anxiety/depression on a scale of 1–3, with 1 indicating no problems/limitations and 3 indicating severe limitation.

### EORTC QLQ C-30 questionnaire

The EORTC QLQ C-30 questionnaire has 30 questions that cover 15 HQL parameters that can be grouped as five functional scales (physical, role, cognitive, emotional and social functioning), three symptom scales (fatigue, pain and nausea/vomiting), six single-item scales (dyspnoea, appetite loss, constipation, diarrhoea, financial difficulties and insomnia) and global health status. All the items were scored on a four-point scale ranging from 1–4 with 1 = not at all, 2 = a little, 3 = quite a bit, and 4 = very much. These scores are then transformed to a continuous scale from 0–100, with a higher score of the functional and global health status designating a better QOL and a higher score of the symptom scales meaning a higher symptomatology.

A clinically important difference was designated when there was a 10-point difference between the scores at different time-points [12].

For this study a validated Italian translation of the questionnaire was used after seeking permission from EORTC. The questionnaires were handed out to the patients as paper handouts before start of therapy and at the end of treatment by the nurse, and were collected the following day. The patients were required to complete the questionnaires themselves.

### Statistical analysis

Continuous variables were expressed as mean ± SD (range). Scoring was performed as per the instructions in the EORTC scoring manual. The statistical software program SPSS version 14 (SPSS Inc., Chicago) was used for data analysis. The difference between pre- and end-of-treatment scores was assessed by using the Mann Whitney test.

## RESULTS

Characteristics of the patients included in this study are depicted in Table 1.

**Table 1.** Patient characteristics

Gender	8 males; 9 females
Age	49.5 ± 16.4
	years(21–73)
Histology	Chordoma 77% (13)
	Chondrosarcoma 23% (4)
Location/Site	Skull Base 70.5% (12)
	Sacrum/paraspinal 29.4% (5)
Prior surgery	Mean 1; Min–Max (0–4)
Prior radiotherapy	None
Occupation	Employed 59% (10)
	Unemployed 12% (2)
	Retired 29% (3)
Marital status	Living with partner 59% (10)
	Living alone 41% (7)
Children	Yes 53% (9)
	No 47% (8)

### Physician-scored pre-treatment status

When assessed prior to start of treatment most of the patients were categorized into the ‘no problem’ group for all parameters *viz.* mobility, self-care, pain/discomfort and anxiety/depression. Only 6–29% of patients were assessed to have some problem, while 12% were scored as having severe limitations (Table 2).

**Table 2.** Physician-assessed pre-treatment scores

Parameter	No problem	Some problem	Severe limitation
Mobility	82% (14)	6% (1)	12% (2)
Self-care	82% (14)	6% (1)	12% (2)
Pain/discomfort	70% (12)	18% (3)	12% (2)
Anxiety/depression	59% (10)	29% (5)	12% (2)

### Global health status

The mean ± SD pre- and post-treatment scores were 71 ± 24.5 and 68.1 ± 18.6, respectively. The mean scores were neither statistically significant nor clinically important (Table 3).

**Table 3.** Pre-treatment versus end-of-treatment mean scores

	Pre-treatment	End-of-treatment	*P*-value	Clinically important difference
Global health status	71 ± 24.5	68.1 ± 18.6	0.5	No
Physical functioning	80.3 ± 30.5	81.2 ± 28.8	0.9	No
Role functioning	82.3 ± 29.7	77.4 ± 32.2	0.1	No
Emotional functioning	77.4 ± 32.1	85.7 ± 24.8	0.09	No
Cognitive functioning	84.3 ± 31.4	88.2 ± 21.9	0.2	No
Social functioning	78.4 ± 34.7	79.4 ± 30.4	0.7	No
Fatigue	28.7 ± 30.4	32.8 ± 30.9	0.4	No
Pain	10.7 ± 18.5	11.7 ± 16.4	0.9	No
Sleep	27.4 ± 35.8	19.6 ± 26.5	0.2	No
Financial difficulty	23.5 ± 28.3	33.33 ± 33.33	0.2	Yes

### Functional scores

The mean ± SD pre-treatment scores for physical, role, emotional, cognitive and social functioning were 80.3 ± 30.5, 82.3 ± 29.7, 77.4 ± 32.1, 84.3 ± 31.4, 78.4 ± 34.7, while the post-treatment scores were 81.2 ± 28.8, 77.4 ± 32.2, 85.7 ± 24.8, 88.2 ± 21.9, 79.4 ± 30.4, respectively (Table 3). These differences were neither statistically significant nor found to be clinically important.

When data was assessed with stratification in the form of no change (no change between pre- and post-treatment scores), trivial change (<4 points change), small difference (4–10 points change), and clinically important difference (>10 points change) for each category individually, clinically important differences were detected in all domains of QOL (Table 4).

**Table 4.** Changes in quality-of-life domains

	No change	Trivial (<4)		Small (4–10)		Medium/Clinically important difference (>10)	
		Improve	Deteriorate	Improve	Deteriorate	Improve	Deteriorate
Global	6% (1)	0	0	12% (2)	24% (4)	24% (4)	35% (6)
Physical	29% (5)	0	0	18% (3)	24% (4)	18% (3)	12% (2)
Role	71% (12)	0	0	0	0	6% (1)	24% (4)
Emotional	47% (8)	0	0	18% (3)	6% (1)	24% (4)	6% (1)
Cognitive	77% (13)	0	0	0	0	17% (3)	6% (1)
Social	59% (10)	0	0	0	0	17% (3)	23% (4)
Fatigue	41% (7)	0	6% (1)	0	0	18% (3)	35% (6)
Pain	65% (11)	0	0	0	0	12% (2)	24% (4)
Sleep	41% (7)	0	0	0	0	35% (6)	24% (4)
Financial difficulty	65% (11)	0	0	0	0	12% (2)	24% (4)

### Symptom scores

Given the location of the tumour, the symptoms were primarily skeletal and neurological. Subsequently, fatigue, pain, insomnia and financial difficulty were chosen for reporting here, as the remaining symptoms like nausea/vomiting, dyspnoea, constipation, diarrhoea and loss of appetite were not encountered in this particular group of patients. The mean ± SD pre-treatment scores for fatigue, pain, insomnia and financial difficulty were 28.7 ± 30.4, 10.7 ± 18.5, 27.4 ± 35.8 and 23.5 ± 28.3, while post-treatment scores were 32.8 ± 30.9, 11.7 ± 16.4, 19.6 ± 26.5 and 33.33 ± 33.33. None of the changes in these parameters were statistically significant, however when individual patient data was analysed, a clinically important improvement in fatigue, pain, sleep and financial difficulty was documented in 18%, 12%, 35% and 12% of the patients, respectively, while clinically important deterioration for the same was documented in 35%, 24%, 24% and 24% of cases, respectively (Tables 3 and 4). The statistical significance of this could not be assessed due to the very low number of events.

### Toxicities

The maximal acute site-specific toxicities recorded in the patients are as shown in Tables 5 and 6. As seen by the data in the tables, the total number of events reported was few and the highest toxicity recorded was Grade II.

**Table 5.** Skull base treatment: maximal toxicity recorded and number of patients [25]

	Grade 1	Grade II	Grade III	Grade IV
Vomiting	1	1	0	0
Nausea	2	1	0	0
Fatigue	0	0	0	0
Headache	5	0	0	0
Hypersomnia	0	0	0	0
Dermatitis	11	1	0	0
Superficial soft tissue fibrosis	0	0	0	0
Oral mucositis	2	1	0	0
Dysphagia	0	0	0	0

**Table 6.** Sacral and spinal treatments: maximal toxicity recorded and number of patients [25]

	Grade I	Grade II	Grade III	Grade IV
Dermatitis	4	2	0	0
Superficial soft tissue fibrosis	0	0	0	0
Proctitis	0	0	0	0
Cystitis	0	0	0	0
Rectal pain	0	0	0	0
Nausea	0	0	0	0
Diarrhoea	0	0	0	0

## DISCUSSION

To best of our knowledge this is the first study to assess quality-of-life parameters in patients with chordomas/chondrosarcomas treated with protons. Additionally, no comparable data on QOL in these patients has been reported from the photon therapy world. We found that QOL parameters are maintained during treatment.

Given the location and natural history of these tumours, multiple surgeries are often needed for maximal surgical resection, and the potential toxicity associated with repeated excisions in areas like the base of skull can lead to considerable morbidity with a rate of 23–33% for new neurological injuries and 9–10% for cerebrospinal fluid leakage [13, 14]. Radical resection is difficult to achieve, consequently debulking or partial resections are usually performed. The standard for primary treatment is therefore a combination of surgery and radiotherapy [15] to improve the local control rate. In our set of patients surgery was performed in all except one case of sacral chordoma that was assessed to be unresectable, in which case only a biopsy was taken.

These tumours are classically considered radioresistant [16–18], and the use of radiation has been debated. However, with improved techniques of delivering radiation, higher doses can be delivered and local control rates can be improved. Protons have an advantage in such scenarios on account of their dose deposition characteristics, and can deposit high doses to the target while respecting the tolerance doses of the surrounding normal organs [19]. It is expected that sparing of the normal tissues can decrease the incidence and severity of the toxicities, thereby helping maintain a good QOL.

Use of QOL measurement in the clinical oncological setting has been shown to improve patient–clinician communication and to be associated with improved QOL and emotional functioning of the patients [20]. The QOL questionnaire can be used as a communication aid with which patients can describe their side effects to clinicians, and it has been demonstrated by Detmar *et al.* [2] as facilitating patient–clinician communication and improving clinician awareness of patient QOL issues. It provides the patients' perspective, and it has been shown to differ from physician-evaluated assessments [21]. This fact was clearly depicted in the discordance seen between the assessments of physical activity status by the patients and by the physicians. As per the observation of the physician in charge, 82% of the patients had no problems with mobility and could take care of themselves without effort, and the evaluation continued to be the same during the course of treatment. From the patients' point of view, 29% of the patients self-assessed their physical functioning as not having changed during the course of treatment, while 18% reported improvement and 12% reported deterioration in the physical functioning score that was deemed to be clinically significant, even though not statistically significant. Similarly, for emotional functioning, 47% of the patients reported no change in status during the course of treatment, 24% reported improvement and 6% reported deterioration. This phenomenon can be attributed to the psychological impact of undergoing treatment itself, especially in cases that had been previously stated to be unresectable or which had large residuals after surgery and no other option available for further treatment. When proton beam therapy was offered as a treatment in such difficult scenarios, patient's anxiety and uncertainty regarding the disease process was decreased, which may have been reflected in improved emotional functioning scores.

Fatigue is a common problem encountered in patients of cancer who undergo radiotherapy [22, 23]. It has been documented as one of the three most negative items affecting QOL in cancer patients. In this subset of patients, no change in fatigue scores was recorded by 41%, worsening was reported in 35%, while improvement was noted in 18% of the patients when comparing the end-of-treatment scores with the pre-treatment scores. As seen in the PARSPORT trial, intensity-modulated radiotherapy (IMRT) is associated with increased fatigue scores compared with conventional irradiation, and this can result in deterioration of the general wellbeing of the patients undergoing IMRT [24]. Despite changes in fatigue scores reported in this study group, no significant impact on the HQL was found. These are slow-responding tumours and are not known to change during the course of treatment. Consequently, symptoms associated with the disease resolve gradually. In keeping with this, pain scores did not change in 65%, deteriorated in 24%, and improved in 12% of the cases. Difficulty in sleeping was improved in 35%, worsened in 24%, and not changed in 41% of patients towards the end of treatment. The improvement could be attributed to decreased anxiety or decreased pain/symptoms.

The other major problem that was cited by the patients was ‘financial difficulty’, even though the patient was not paying for the treatment, as they had been treated in the experimental phase of the machine according to the rules of the Italian Health Ministry. The probable reason for this was the costs incurred by the patient when living away from their home during the duration of the treatment (carried out on an out-patient basis).

Apart from global health status and physical functioning scores, there was no change in the majority of cases with respect to the remaining functional domains/symptom scores when comparing pre- versus end-of-treatment values. When comparing the clinically important differences, the results were very heterogeneous, with some patients improving and others deteriorating. It is too early to draw a firm conclusion from this scenario, given the short follow-up and the small sample. As mentioned, these are slow-responding tumours and there is a possibility that late effects may have an impact on the HQL later.

The treatments were well tolerated by all patients and the maximum toxicities scored as per CTCAE version 3 were Grade 2, as depicted in Tables 5 and 6. These have been discussed in detail by Tuan *et al.* [25] in this journal. The most common toxicity reported was dermatitis, which is in keeping with the fact that protons lack the skin-sparing effect associated with photons. There were no interruptions in treatment due to toxicities.

Patient-reported outcomes like QOL are subjective and can be affected by a number of factors, both treatment and non-treatment related. Though it was not possible to carry out a statistical correlation of QOL with acute toxicity scores due to the small sample size, the low incidence and severity of acute side events reported with the proton treatment suggests that such treatments are very well tolerated. It can be safely presumed that QOL is maintained during treatment despite the lack of statistically significant data and the small sample size.

## CONCLUSION

Assessment of QOL forms an integral part of patient-reported outcome measures. This type of study in chordoma/chondrosarcoma patients treated with proton beam therapy is unique. We were unable to find any such similar study in the literature, thus ruling out the possibility of comparison with similar studies.

It is too early to draw firm conclusions regarding the changes in HQL with proton therapy treatment. As these are slow-responding tumours, longer follow-ups may reveal additional information regarding HQL changes. Consequently it will be essential to re-evaluate QOL in these patients at repeated intervals. This is an ongoing study at CNAO and results will be updated at a later date. However, it can be safely concluded that QOL scores were at the very least maintained, if not improved, in the majority of the patients during the course of proton beam therapy.
